# Huanglian-Wendan Decoction Inhibits NF-*κ*B/NLRP3 Inflammasome Activation in Liver and Brain of Rats Exposed to Chronic Unpredictable Mild Stress

**DOI:** 10.1155/2018/3093516

**Published:** 2018-04-29

**Authors:** Ke-Ke Jia, Hong Ding, Han-Wen Yu, Tie-Jun Dong, Ying Pan, Ling-Dong Kong

**Affiliations:** State Key Laboratory of Pharmaceutical Biotechnology, School of Life Sciences, Nanjing University, Nanjing 210023, China

## Abstract

Depression is a common mental disorder in modern society. A traditional Chinese medicine Huanglian-Wendan decoction with potential anti-inflammation is used as a clinical antidepressant. Our previous study showed central and peripheral inflammatory responses in a rat model of depression developed by chronic unpredictable mild stress (CUMS). Here, we investigated the anti-inflammatory activity and mechanism of Huanglian-Wendan decoction in CUMS rats. LC-MS/MS and HPLC were performed to determine the major compounds in water extract of this decoction. This study showed that Huanglian-Wendan decoction significantly increased sucrose consumption and reduced serum levels of interleukin-1 beta (IL-1*β*), IL-6, and alanine aminotransferase (ALT) in CUMS rats. Moreover, this decoction inhibited nuclear entry of nuclear factor-kappa B (NF-*κ*B) with the reduction of phosphorylated protein of NF-*κ*B (p-NF-*κ*B) and inhibitor of NF-*κ*B alpha (p-I*κ*B*α*) and downregulated protein of nod-like receptor family pyrin domain-containing 3 (NLRP3), apoptosis-associated speck-like protein containing CARD (ASC), cysteinyl aspartate-specific proteinase-1 (Caspase-1), and IL-1*β* in liver and brain regions of CUMS rats. These findings demonstrated that Huanglian-Wendan decoction had antidepressant activity with hepatoprotection in CUMS rats coinciding with its anti-inflammation in both periphery and central. The inhibitory modulation of NF-*κ*B and NLRP3 inflammasome activation by Huanglian-Wendan decoction may mediate its antidepressant action.

## 1. Introduction

Depression is a complex psychiatric disorder with a disabling medical condition. Recently, the World Health Organization informs that there are over 300 million people worldwide suffering from depression, causing high health loss [1]. Although the underlying molecular mechanism of depression pathology remains elusive, compelling evidence suggests that inflammation may play a critical role [2]. Inflammation molecule, especially nuclear factor-kappa B (NF-*κ*B), levels in plasma are increased in patients with major depression [3]. More importantly, neuroinflammation is observed in patients with major depressive episodes [4]. Nod-like receptor family pyrin domain-containing 3 (NLRP3) inflammasome, a multiprotein complex consisting of NLRP3, apoptosis-associated speck-like protein containing CARD (ASC), and cysteinyl aspartate-specific proteinase-1 (Caspase-1) controlling the release of interleukin-1 beta (IL-1*β*), is strongly recognized to develop depression [5]. NLRP3 inflammasome activation is observed in mononuclear blood cells of patients with major depression [6] and in brain of chronic unpredictable mild stress- (CUMS-) induced depression in rats by us [7]. In fact, the CUMS procedure can develop a number of behavioral and physiological alteration in rodents, being similar to depression symptom of clinical patients [8]. This animal model has been suggested to be a potentially reliable model to investigate depression-associated neuroinflammation [7, 9].

The results from our and others' studies indicate the activation of the NF-*κ*B pathway and NLRP3 inflammasome in brain regions prefrontal cortex [7, 10–12] and hippocampus [12–14] in rodents of CUMS-induced depression, which are consistent with the reports in depressed patients [3, 6]. As a clinical routine index of liver function, alanine aminotransferase (ALT) levels in blood are increased in patients with major depression [15], thus being used to predict new onset of depression in subjects with health screening examination [16]. Consistently, the CUMS procedure increases serum levels of ALT in rats [12, 17]. In fact, the CUMS procedure alters hepatic metabolic profiles (such as reduction of carnitine) in rats [17] and proteomic profiles (such as proteins related to inflammatory response, immune regulation, and NF-*κ*B signaling network) in mice [18] and activates hepatic NLRP3 inflammasome in rats [12]. These observations suggest that NF-*κ*B and NLRP3 inflammasome activation in liver and brain regions may contribute to depression and provide a novel anti-inflammatory strategy for treatment of depression.

Most of the prescribed depression medications are designed to target neurotransmitters and have some common side effects, such as headache, dry mouth, insomnia, and sexual dysfunction [19]. Interestingly, some of them possess anti-inflammatory properties. For example, fluoxetine inhibits proinflammatory cytokine production in lipopolysaccharide-stimulated microglial cells [20]. Recently, nonsteroidal anti-inflammatory drugs show potential for treating some mental disorders, and mefenamic acid with inhibitory effects on NLRP3 inflammasome activation is reported to protect against memory loss induced by amyloid beta and in a transgenic mouse model of Alzheimer's disease [21]. Thus, it is necessary to find some novel, efficacious therapeutic agents with anti-inflammation for the treatment of depression.

Huanglian-Wendan decoction is a traditional Chinese medicine (TCM), consisting of *Coptis chinensis Franch*, *Pinellia ternata (Thunb.) Breit*, *Citrus reticulata Blanco*, *Citrus aurantium L.*, *Poria Cocos (Schw) Wolf*, *Bambusa tuldoides Munro*, *Glycyrrhiza uralensis Fisch*, and *Zingiber officinale Roscoe*. It is originated from the formula of Wendan decoction and first recorded in a book called “Liu-Yin-Tiao-Bian” written by Ting-Zhen Lu in the Qing dynasty. In TCM theory, depression is linked with *Liver-Qi* stagnation, while soothing the *Liver* is an effective strategy to treat depression. Huanglian-Wendan decoction has the function of heat-clearing, damp-drying, qi-regulating, and phlegm-eliminating, resulting in soothing the *Liver*, promoting the *Gallbladder*, and harmonizing the *Stomach*. Huanglian-Wendan decoction is clinically used to treat anxiety disorders [22], liver stagnation, and phlegm-type generalized anxiety [23] and depression [24]. Moreover, this decoction is reported to reduce serum interleukin-6 (IL-6) levels in rats with metabolic syndrome [25] and inhibit the release of IL-1*β*, IL-6, and tumor necrosis factor-*α* (TNF-*α*) in the hippocampus of rats with diabetic encephalopathy [26], indicating that Huanglian-Wendan decoction has anti-inflammatory activity.

In the present study, we investigated the antidepressant effect of Huanglian-Wendan decoction and its possible underlying mechanism in CUMS rats. Specifically, we evaluated its anti-inflammatory activity on NF-*κ*B and NLRP3 inflammasome activation in the liver and brain regions of CUMS rats.

## 2. Materials and Methods

### 2.1. Preparation of Huanglian-Wendan Decoction

Huanglian-Wendan decoction was prepared from eight herbs according to the original formula in “Liu-Yin-Tiao-Bian.” All these herbs were purchased from Medicinal Materials Co. of Jiangsu Province (Nanjing, P. R. China), and the origin and batch number of all herbs are listed in Table 1. The raw herbs were preimmersed in eight-time volumes of distilled water for 30 min then boiled for 60 min. Thereafter, four-time volumes of distilled water were added into the residue, decocted twice for 30 min. All the collected supernatants were filleted through eight layers of gauze, condensed into extractum by rotary evaporators.

### 2.2. Component Analysis of Huanglian-Wendan Decoction

The qualitative analysis of components in water extract of Huanglian-Wendan decoction was employed by liquid chromatography-tandem mass spectrometry (LC-MS/MS). The liquid chromatography equipment consisted of a Shimadzu LC-30AD pump and a Shimadzu SPD-20A detector. And the accurate mass spectrometric experiments were operated in the positive and negative ion mode of a TripleTOF 4600 system with a DuoSpray ion source (AB Sciex, California, USA). 1086 mg of Huanglian-Wendan decoction extract was dissolved in 10 mL solvent (water : ethanol = 1 : 1), filtered through a 0.22 *μ*m membrane filter, and then centrifuged at 16,000 ×g for 5 min; the supernatant was collected for analysis. Analysis was carried out on a C_18_ column (150 mm × 4.6 mm, 3 *μ*m), and the column temperature was maintained at 40°C. The mobile phase was composed of A (0.1% formic acid in water, *v*/*v*) and B (acetonitrile) using a gradient elution of 5% B at 0–2 min, 5–50% B at 2–30 min, 50–90% B at 30–33 min, 90% B at 33–37 min, and 90–5% B at 37–37.1 min. The injection volume for the sample was 5 *μ*L. The flow rate was 0.4 mL/min. The operation parameters were as follows: curtain gas, 30 psi; ion source gas 1 and ion source gas 2, 55 psi; ion spray voltage floating, 5500 V; temperature, 550°C; collision energy, 40 V; and collision energy spread, 20 V. Data was managed with PeakView software (AB Sciex, California, USA). MS^2^ fingerprints of components in Huanglian-Wendan decoction water extract were referred to some databases like Metlin, SDBS, and MassBank and some references [27–29] for preliminary confirmation. Quantitative analysis of the above components in Huanglian-Wendan decoction water extract was employed by high-performance liquid chromatography (HPLC) with a diode array detector (Agilent Technologies 1200 Series, USA) using an external method. Analysis was carried out on a C_18_ column (250 mm × 4.6 mm, 5 *μ*m), and the column temperature was maintained at 30°C. The mobile phase was composed of A (0.1% formic acid in water, *v*/*v*) and B (acetonitrile) using a gradient elution of 5% B at 0–2 min, 5–30% B at 2–10 min, 30–90% B at 10–20 min, 90% B at 20–35 min, and 90–5% B at 35–35.1 min. The flow rate was set at 0.8 mL/min. The injection volume for the sample was 2 *μ*L, and that for reference standards was 3 *μ*L. The monitoring wavelength was set at 210 and 280 nm. Ferulic acid (1.27 mg), naringin (1.97 mg), hesperidin (1.57 mg), neohesperidin (2.03 mg), berberine (2.70 mg), palmatine (2.23 mg), limonin (1.56 mg), and glycyrrhizic acid (2.83 mg) were weighted, respectively. Ferulic acid (no. 110773), naringin (no. 110722), hesperidin (no. 110721), neohesperidin (no. 111857), berberine (no. 110713), palmatine (no. 110732), limonin (no. 110800), and glycyrrhizic acid (no. 110731) were purchased from National Institutes for Food and Drug Control (Beijing, P. R. China). Subsequently, these reference standards were dissolved in 10 mL solvent (water : ethanol = 1 : 1), and centrifuged at 16,000 ×g for 5 min, then the supernatant was collected for analysis. Finally, the content of each component in Huanglian-Wendan decoction water extract was calculated by the following formula:

(1) $$\begin{array}{c} \frac{standard\;concentration\;\left(mg/mL\right)\times standard\;injection\;volume\;\left(µL\right)\times sample\;peak\;area\;\left(mAU\times s\right)}{sample\;concentration\;\left(g/mL\right)\times sample\;injection\;volume\;\left(µL\right)\times standard\;peak\;area\;\left(mAU\times s\right)} \end{array}$$

### 2.3. Animal Experiment Design

Male Wistar rats, weighing 190 to 230 g, were purchased from Beijing Vital River Laboratories (P. R. China, certificate no. SCXK (Jing) 2012–0001). All rats were raised in a controlled temperature and humidity with a 12 h light/dark cycle, except the procedures described below. Rats were allowed to acclimatize for at least one week before the experiment started. All procedures on animals followed the guidelines established by the Institutional Animal Care Committee at the Nanjing University and the China Council on Animal Care at Nanjing University.

### 2.4. Sucrose Consumption Test and Experimental Groups

Rats were individually trained to consume 1% sucrose solution (*w*/*v*) before the CUMS procedure. A sucrose solution consumption test was performed after an 18 h period of food and water deprivation by the offering of sucrose solution for a 1 h test (11 : 00–12 : 00). Subsequently, a sucrose consumption test was employed every two weeks at 12 : 00–13 : 00 on Friday under similar conditions for a total of 12 weeks. After the baseline test of sucrose consumption, rats were randomly divided into non-CUMS (*n* = 12) and CUMS (*n* = 48) groups. The non-CUMS rats were kept in a separate room with normal care, while the CUMS rats were exposed to a series of stressors: water or food deprivation, empty water bottles, stroboscopic light, intermittent white noise, and illumination overnight. All of those stressors were employed randomly, constantly, and nonrepetitively within a total of 12 weeks. After the first six-week CUMS procedure, CUMS rats were divided into four subgroups: CUMS-vehicle (water, 5 mL/kg, *n* = 12); Huanglian-Wendan decoction (5690 mg/kg, *n* = 12), Huanglian-Wendan decoction (11,380 mg/kg, *n* = 12), and fluoxetine (10 mg/kg, *n* = 12).

### 2.5. Drug Administration

The daily human dosage of Huanglian-Wendan decoction formula from “Liu-Yin-Tiao-Bian” is 86 g (total raw materials). This daily dose of the basic and modified Huanglian-Wendan decoction has been used clinically in anxiety disorders, liver stagnation, and phlegm-type generalized anxiety and depression [22–24]. By dose translation from human to animals using the body surface area normalization method, the daily dose of Huanglian-Wendan decoction for rat is 5690 mg (total raw materials)/kg•body weight. And the daily doses of Huanglian-Wendan decoction with neuronal activity in animal studies are about 3000–12,000 mg (total raw materials)/kg•body weight of rat [26, 30]. Thus, the daily doses of 5690 and 11,380 mg (total raw materials)/kg•body weight for Huanglian-Wendan decoction were chosen in the present study, which were equivalent to doses of 2714 mg and 5428 mg (extract)/kg•body weight converted by the extraction yield of 47.7%. Additionally, Huanglian-Wendan decoction water extract and fluoxetine were dissolved in distilled water to concentrations of 543 mg/mL, 1086 mg/mL, and 2 mg/mL for application, respectively, as rats were administrated by gavage in a volume of 5 mL/kg for 6 weeks at 12 : 00–13 : 00 once daily.

### 2.6. Serum and Tissue Collection

After the final drug treatment, all rats were fasted for 16 h, anaesthetized by sodium pentobarbital, and sacrificed immediately after blood collection. Blood samples were collected from the carotid artery and centrifuged at 3000 ×g, 4°C, for 10 min to get serum. The liver, prefrontal cortex, hippocampus, and hypothalamus tissues of rats were rapidly removed on an ice-cold plate, then quickly transferred into liquid nitrogen and kept at −80°C for future analysis.

### 2.7. Measurement of Serum Levels of IL-1*β*, IL-6, and ALT

Serum levels of IL-1*β* were performed using an enzyme-linked immune sorbent assay (ELISA) kit purchased from R&D Systems (RLB00, USA), those of IL-6 were measured using an ELISA kit purchased from ExCell Biotechnology (ER-003, P. R. China), and those of ALT were measured by a commercial kit obtained from Nanjing Jiancheng Bioengineering Institute (C009-2, P. R. China), according to the manufacturer's instructions, respectively.

### 2.8. Western Blot Analysis

The total, cytoplasmic, and nuclear protein samples of rat liver, prefrontal cortex, hippocampus, and hypothalamus tissues were extracted by corresponding methods, respectively. The tissues were first homogenized with RIPA lysates containing phenylmethanesulfonyl fluoride (PMSF), then centrifuged at 12,000 ×g, 4°C, for 20 min to get the supernatant. The protein concentrations of the supernatant were assayed by the BCA assay kit (Thermo Scientific, USA), and these samples were made into total protein extraction for Western blot analysis. The cytoplasmic and nuclear protein samples for NF-*κ*B assay were extracted by a commercial kit purchased from Beyotime Biotechnology (P0013, P. R. China) according to the manufacturer's instructions. Briefly, tissues were homogenized with cytoplasmic protein extraction reagent, vortex, and centrifuged at 15,000 ×g, 4°C, for 5 min to collect the supernatant (cytoplasmic protein). Then precipitate were re-suspended with nuclear protein extraction reagent, vortex and centrifuged at 15,000 ×g, 4°C for 10 min to get the supernatant (nuclear protein). All protein samples were separated by SDS-polyacrylamide gel electrophoresis (SDS-PAGE) and subsequently transferred into polyvinylidene fluoride (PVDF) membranes (Millipore Corporation, USA). Then the membranes were blocked with 5% skim milk and incubated with corresponding primary antibodies at 4°C for overnight. Membranes were washed with Tris-buffered saline and Tween (TBST), then incubated with horseradish peroxidase-conjugated secondary antibody for 1 h and thereafter washed with TBST for 1 h. Subsequently, the membranes were incubated with ECL reagent (Thermo Scientific, USA) for 2 min and exposed with an X-ray film. Finally, gray values of all samples were measured by ImageJ software (National Institutes of Health, USA) and relative quantitation was calculated by normalized to lamin A/C (for nuclear protein) or GAPDH, *β*-actin, or *β*-tubulin (for cytoplasmic and total protein), respectively. Primary antibodies included antibodies of anti-p-NF-*κ*B (#3033, 1 : 1000, CST, USA), anti-NF-*κ*B (#8242, 1 : 1000, CST, USA), anti-p-I*κ*B*α* (#2859, 1 : 1000, CST, USA), anti-I*κ*B*α* (#4814, 1 : 1000, CST, USA), anti-NLRP3 (#13158, 1 : 1000, CST, USA), anti-ASC (#13833, 1 : 1000, CST, USA), anti-Caspase-1 (sc-514, 1 : 500, Santa Cruz, USA), anti-IL-1*β* (MAB-5011, 1 : 500, R&D Systems, USA), anti-lamin A/C (#4777, 1 : 1000, CST, USA), anti-GAPDH (sc-25778, 1 : 1000, Santa Cruz, USA), anti-*β*-actin (#4970, 1 : 1000, CST, USA), and anti-*β*-tubulin (#2128, 1 : 1000, CST, USA). Secondary antibodies were incubated with goat anti-rabbit IgG (#7074, 1 : 3000, CST, USA) and goat anti-mouse IgG (sc-2005, 1 : 5000, Santa Cruz, USA).

### 2.9. Statistical Analysis

All data were expressed as mean ± S.E.M. The statistical analysis system (GraphPad Prism 6, USA) with one-way ANOVA followed by LSD post hoc test was applied to analyze the data and obtain the figures. Statistical significance was set at if *P* < 0.05.

## 3. Results

### 3.1. Qualitative and Quantitative Analysis of Components in Water Extract of Huanglian-Wendan Decoction

**Figure 1:**
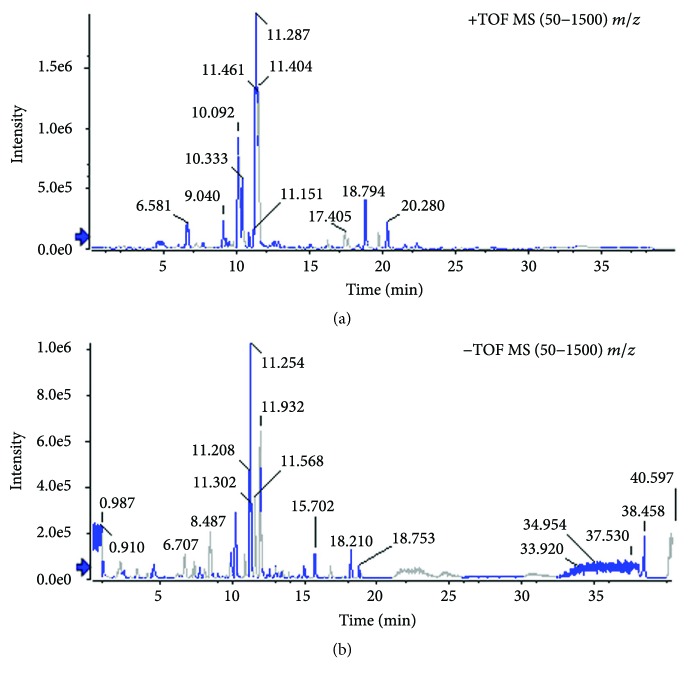
Profile of base peak chromatograph in water extract of Huanglian-Wendan decoction. (a) Positive ion mode and (b) negative ion mode were detected by LC-MS/MS.

Qualitative analysis of compounds in water extract of Huanglian-Wendan decoction was carried out by LC-MS/MS in positive ion mode (Figure 1(a) and Table 2) and negative ion mode (Figure 1(b) and Table 3). We identified 31 compounds in the positive ion mode and 20 compounds in the negative ion mode. According to the quality control of each herb in Pharmacopoeia of the People's Republic of China (2015 version), 8 major compounds in water extract were chosen for quantitative analysis (Table 4). After calculation, this water extract contained 3.39 mg/g of ferulic acid, 11.74 mg/g of naringin, 21.08 mg/g of hesperidin, 21.66 mg/g of neohesperidin, 2.31 mg/g of berberine, 6.37 mg/g of palmatine, 0.23 mg/g of limonin, and 1.71 mg/g of glycyrrhizic acid, respectively.

### 3.2. Huanglian-Wendan Decoction Attenuates Depressive-Like Behavior in CUMS Rats

Sucrose solution consumption reduction in CUMS rodents is commonly considered a depressive-like behavior (anhedonia) [31]. As shown in Table 5, after a 2-week CUMS procedure, sucrose consumption was significantly decreased (*P* < 0.001) in CUMS rats compared with non-CUMS rats, and this trend was kept till the end of the 12th week. After the 2-week treatment, Huanglian-Wendan decoction (5690 mg/kg: *P* < 0.01) and fluoxetine (*P* < 0.001) obviously increased sucrose consumption in CUMS rats. Huanglian-Wendan decoction maintained and further improved sucrose consumption in CUMS rats at the 10th week (5690 mg/kg: *P* < 0.001; 11,380 mg/kg: *P* < 0.01) and 12th week (*P* < 0.001). Fluoxetine also improved CUMS-induced depressive-like behavior by increasing sucrose consumption at both 10th and 12th weeks (*P* < 0.001).

### 3.3. Huanglian-Wendan Decoction Decreases Serum Levels of IL-1*β*, IL-6, and ALT in CUMS Rats

**Figure 2:**
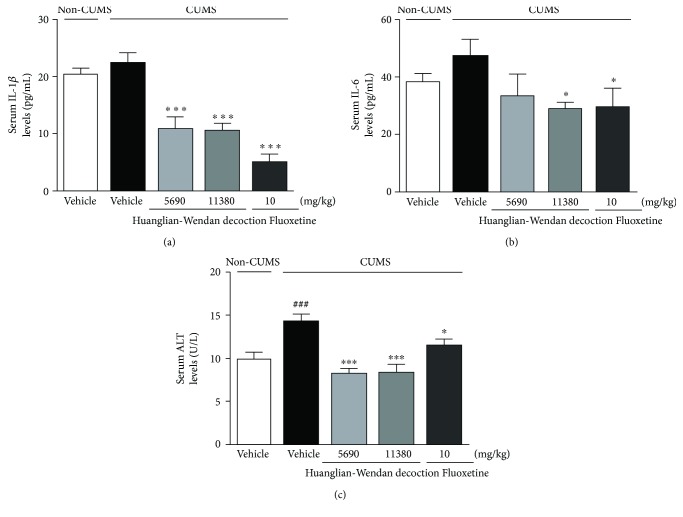
Effects of Huanglian-Wendan decoction on serum IL-1*β*, IL-6, and ALT levels in CUMS rats. (a) Serum IL-1*β* levels were measured by an ELISA assay kit. (b) Serum IL-6 levels were measured by an ELISA assay kit. (c) Serum ALT levels were measured by a commercial assay kit. All data were expressed as mean ± S.E.M. (*n* = 6). ^###^*P* < 0.001 compared with the non-CUMS group; ^∗^*P* < 0.05 and ^∗∗∗^*P* < 0.001 compared with the CUMS group.

Although there was no significant change in serum IL-1*β* or IL-6 levels between the CUMS and non-CUMS groups, Huanglian-Wendan decoction as well as fluoxetine evidently reduced serum levels of IL-1*β* (*P* < 0.001, Figure 2(a)) and IL-6 (Huanglian-Wendan decoction 11,380 mg/kg and fluoxetine: *P* < 0.05, Figure 2(b)) levels in CUMS rats. High serum ALT levels were detected in CUMS rats (*P* < 0.001), which were significantly reversed by Huanglian-Wendan decoction (*P* < 0.001) and fluoxetine (*P* < 0.05) (Figure 2(c)).

### 3.4. Huanglian-Wendan Decoction Blocks Hepatic NF-*κ*B and NLRP3 Inflammasome Activation in CUMS Rats

**Figure 3:**
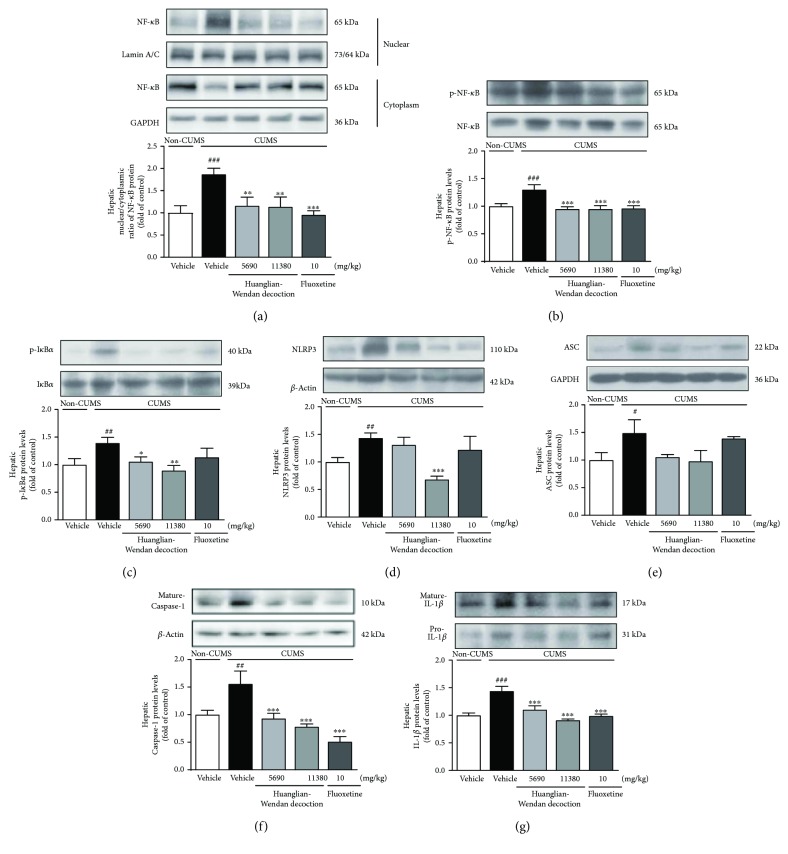
Effects of Huanglian-Wendan decoction on NF-*κ*B and NLRP3 inflammasome activation in liver of CUMS rats. (a) The nuclear entry of NF-*κ*B was quantitated by the nuclear/cytoplasmic ratio of NF-*κ*B protein levels normalized by lamin A/C or GAPDH, respectively. (b) The relative protein levels of p-NF-*κ*B were normalized to NF-*κ*B. (c) The relative protein levels of p-I*κ*B*α* were normalized to I*κ*B*α*. (d) The relative protein levels of NLRP3 were normalized to *β*-actin. (e) The relative protein levels of ASC were normalized to GAPDH. (f) The relative protein levels of mature Caspase-1 were normalized to *β*-actin. (g) The relative protein levels of mature IL-1*β* were normalized to pro-IL-1*β*. Protein levels of p-NF-*κ*B, NF-*κ*B, p-I*κ*B*α*, I*κ*B*α*, NLRP3, ASC, mature Caspase-1, mature IL-1*β*, pro-IL-1*β*, *β*-actin, and GAPDH were analyzed by Western blot method. The data were expressed as fold of control values. All data were expressed as mean ± S.E.M. (*n* = 6–9). ^#^*P* < 0.05, ^##^*P* < 0.01, and ^###^*P* < 0.001 compared with the non-CUMS group; ^∗^*P* < 0.05, ^∗∗^*P* < 0.01, and ^∗∗∗^*P* < 0.001 compared with the CUMS group.

The CUMS procedure induced hepatic nuclear entry of NF-*κ*B (nuclear/cytoplasmic (N/C) ratio of NF-*κ*B, *P* < 0.001, Figure 3(a)) with an increase in protein levels of p-NF-*κ*B (*P* < 0.001, Figure 3(b)) and p-I*κ*B*α* (*P* < 0.01, Figure 3(c)) in rats. Huanglian-Wendan decoction obviously reduced the N/C ratio of NF-*κ*B (*P* < 0.01) and protein levels of p-NF-*κ*B (*P* < 0.001) and p-I*κ*B*α* (5690 mg/kg: *P* < 0.05; 11,380 mg/kg: *P* < 0.01), while fluoxetine significantly reduced the N/C ratio of NF-*κ*B (*P* < 0.001) and p-NF-*κ*B (*P* < 0.001) but slightly decreased p-I*κ*B*α* in the liver of CUMS rats. Moreover, NLRP3 inflamamsome activation was detected in the liver of CUMS rats, as increased protein levels of NLRP3 (*P* < 0.01, Figure 3(d)), ASC (*P* < 0.05, Figure 3(e)), and mature Caspase-1 (*P* < 0.01, Figure 3(f)). Huanglian-Wendan decoction suppressed NLRP3 inflamamsome activation by significantly downregulating protein levels of NLRP3 (11,380 mg/kg: *P* < 0.001) and mature Caspase-1 (*P* < 0.001) in CUMS rats. Fluoxetine markedly decreased hepatic protein levels of mature Caspase-1 (*P* < 0.001). Accordingly, the CUMS procedure induced an increase in hepatic IL-1*β* protein levels (*P* < 0.001, Figure 3(g)), which were restored by Huanglian-Wendan decoction and fluoxetine (*P* < 0.001).

### 3.5. Huanglian-Wendan Decoction Inhibits Central NF-*κ*B and NLRP3 Inflammasome Activation in CUMS Rats

**Figure 4:**
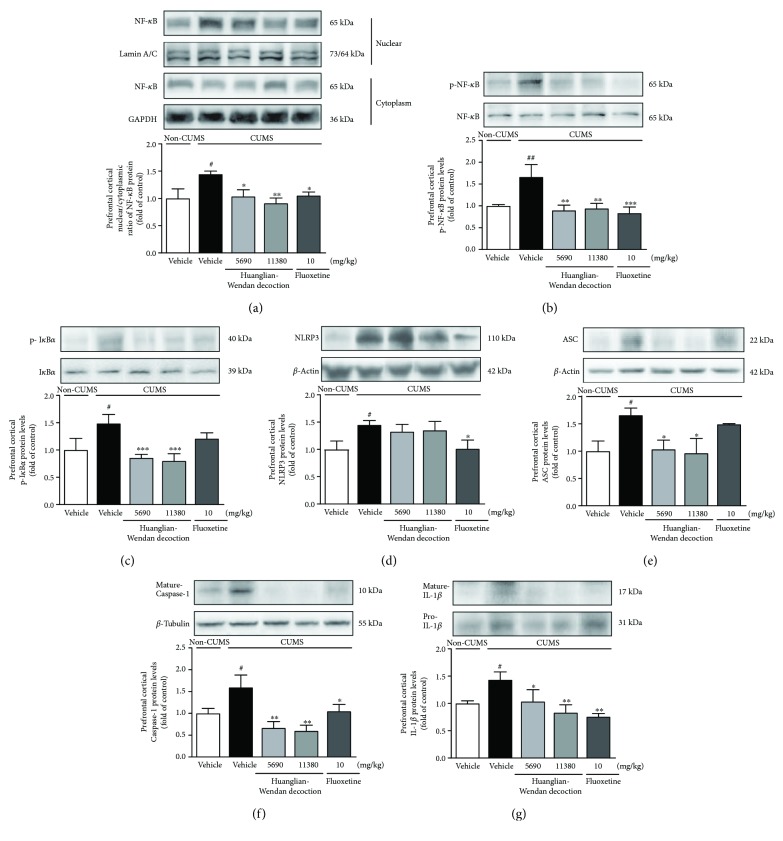
Effects of Huanglian-Wendan decoction on NF-*κ*B and NLRP3 inflammasome activation in the prefrontal cortex of CUMS rats. (a) The nuclear entry of NF-*κ*B was quantitated by the nuclear/cytoplasmic ratio of NF-*κ*B protein levels normalized by lamin A/C or GAPDH, respectively. (b) The relative protein levels of p-NF-*κ*B were normalized to NF-*κ*B. (c) The relative protein levels of p-I*κ*B*α* were normalized to I*κ*B*α*. (d) The relative protein levels of NLRP3 were normalized to *β*-actin. (e) The relative protein levels of ASC were normalized to *β*-actin. (f) The relative protein levels of mature Caspase-1 were normalized to *β*-tubulin. (g) The relative protein levels of mature IL-1*β* were normalized to pro-IL-1*β*. Protein levels of p-NF-*κ*B, NF-*κ*B, p-I*κ*B*α*, I*κ*B*α*, NLRP3, ASC, mature Caspase-1, mature IL-1*β*, pro-IL-1*β*, *β*-actin, and *β*-tubulin were analyzed by the Western blot method. The data were expressed as fold of control values. All data were expressed as mean ± S.E.M. (*n* = 4–8). ^#^*P* < 0.05 and ^##^*P* < 0.01 compared with non-CUMS group; ^∗^*P* < 0.05, ^∗∗^*P* < 0.01 and ^∗∗∗^*P* < 0.001 compared with the CUMS group.

**Figure 5:**
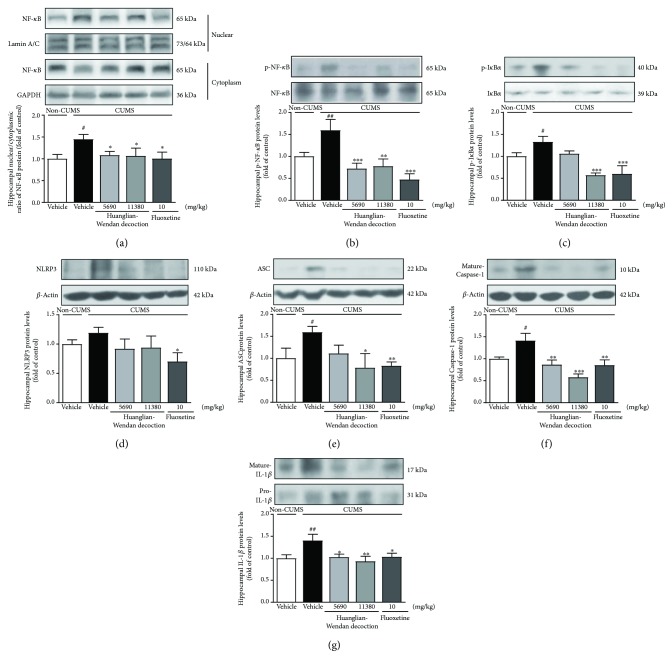
Effects of Huanglian-Wendan decoction on NF-*κ*B and NLRP3 inflammasome activation in the hippocampus of CUMS rats. (a) The nuclear entry of NF-*κ*B was quantitated by the nuclear/cytoplasmic ratio of NF-*κ*B protein levels normalized by lamin A/C or GAPDH, respectively. (b) The relative protein levels of p-NF-*κ*B were normalized to NF-*κ*B. (c) The relative protein levels of p-I*κ*B*α* were normalized to I*κ*B*α*. (d) The relative protein levels of NLRP3 were normalized to *β*-actin. (e) The relative protein levels of ASC were normalized to *β*-actin. (f) The relative protein levels of mature Caspase-1 were normalized to *β*-actin. (g) The relative protein levels of mature IL-1*β* were normalized to pro-IL-1*β*. Protein levels of p-NF-*κ*B, NF-*κ*B, p-I*κ*B*α*, I*κ*B*α*, NLRP3, ASC, mature Caspase-1, mature IL-1*β*, pro-IL-1*β*, and *β*-actin were analyzed by the Western blot method. The data were expressed as fold of control values. All data were expressed as mean ± S.E.M. (*n* = 4–8). ^#^*P* < 0.05 and ^##^*P* < 0.01 compared with the non-CUMS group; ^∗^*P* < 0.05, ^∗∗^*P* < 0.01, and ^∗∗∗^*P* < 0.001 compared with the CUMS group.

**Figure 6:**
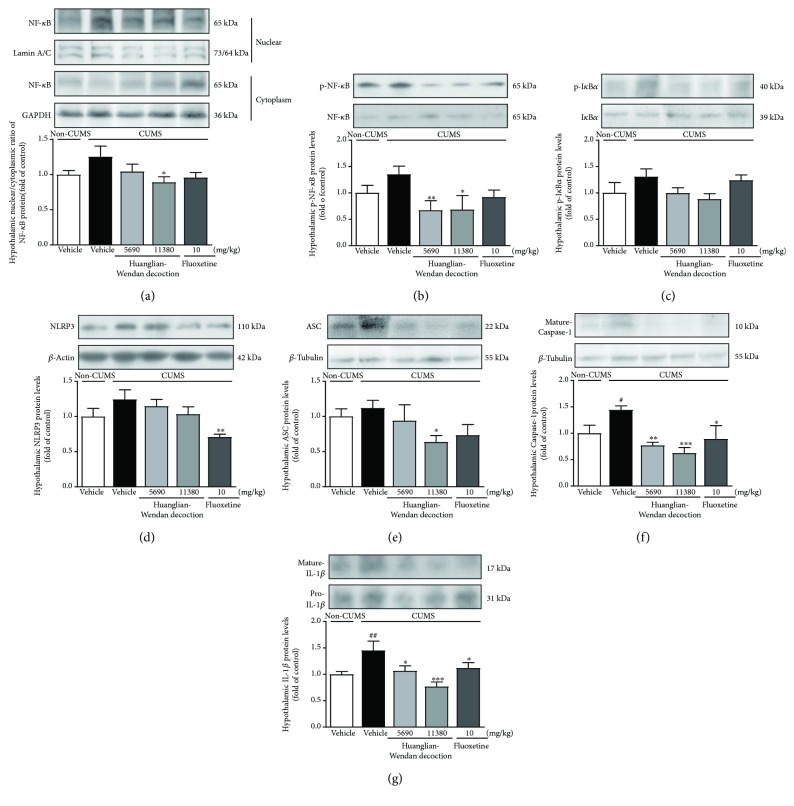
Effects of Huanglian-Wendan decoction on NF-*κ*B and NLRP3 inflammasome activation in the hypothalamus of CUMS rats. (a) The nuclear entry of NF-*κ*B was quantitated by the nuclear/cytoplasmic ratio of NF-*κ*B protein levels normalized by lamin A/C or GAPDH, respectively. (b) The relative protein levels of p-NF-*κ*B were normalized to NF-*κ*B. (c) The relative protein levels of p-I*κ*B*α* were normalized to I*κ*B*α*. (d) The relative protein levels of NLRP3 were normalized to *β*-actin. (e) The relative protein levels of ASC were normalized to *β*-tubulin. (f) The relative protein levels of mature Caspase-1 were normalized to *β*-tubulin. (g) The relative protein levels of mature IL-1*β* were normalized to pro-IL-1*β*. Protein levels of p-NF-*κ*B, NF-*κ*B, p-I*κ*B*α*, I*κ*B*α*, NLRP3, ASC, mature Caspase-1, mature IL-1*β*, pro-IL-1*β*, *β*-actin, and *β*-tubulin were analyzed by the Western blot method. The data were expressed as fold of control values. All data were expressed as mean ± S.E.M. (*n* = 4–8). ^#^*P* < 0.05 and ^##^*P* < 0.01 compared with the non-CUMS group; ^∗^*P* < 0.05, ^∗∗^*P* < 0.01, and ^∗∗∗^*P* < 0.001 compared with the CUMS group.

Furthermore, central NF-*κ*B and NLRP3 inflammasome were detected to be activated at protein levels in brain regions including prefrontal cortex, hippocampus, and/or hypothalamus in CUMS rats (Figures 4–6).

In the prefrontal cortex, the CUMS procedure increased the N/C ratio of NF-*κ*B (*P* < 0.05) and protein levels of p-NF-*κ*B (*P* < 0.01) and p-I*κ*B*α* (*P* < 0.05), as well as NLRP3 (*P* < 0.05), ASC (*P* < 0.05), and mature Caspase-1 (*P* < 0.05) in rats (Figures 4(a)–4(f)). Huanglian-Wendan decoction attenuated CUMS-induced activation of prefrontal cortical NF-*κ*B and NLRP3 inflammasome by obviously reducing the N/C ratio of NF-*κ*B (5690 mg/kg: *P* < 0.05; 11,380 mg/kg: *P* < 0.01) and protein levels of p-NF-*κ*B (*P* < 0.01), p-I*κ*B*α* (*P* < 0.01), ASC (*P* < 0.05), and mature Caspase-1 (*P* < 0.01) in CUMS rats. Fluoxetine also significantly weakened NF-*κ*B and NLRP3 inflammasome activation by reducing the N/C ratio of NF-*κ*B (*P* < 0.05) and protein levels of p-NF-*κ*B (*P* < 0.001), NLRP3 (*P* < 0.05), and mature Caspase-1 (*P* < 0.05) in CUMS rats. Moreover, the CUMS procedure increased prefrontal cortical IL-1*β* protein levels in rats (*P* < 0.05), which were reversed by Huanglian-Wendan decoction (5690 mg/kg: *P* < 0.05; 11,380 mg/kg: *P* < 0.01) and fluoxetine (*P* < 0.01) (Figure 4(g)), respectively.

In the hippocampus, the CUMS procedure increased the N/C ratio of NF-*κ*B (*P* < 0.05, Figure 5(a)) and protein levels of p-NF-*κ*B (*P* < 0.01, Figure 5(b)) and p-I*κ*B*α* (*P* < 0.05, Figure 5(c)) in rats. Huanglian-Wendan decoction significantly reduced the N/C ratio of NF-*κ*B (*P* < 0.05) and protein levels of p-NF-*κ*B (5690 mg/kg: *P* < 0.001; 11,380 mg/kg: *P* < 0.01) and p-I*κ*B*α* (11,380 mg/kg: *P* < 0.001) in CUMS rats. Fluoxetine significantly inhibited hippocampal NF-*κ*B activation by reducing the N/C ratio of NF-*κ*B (*P* < 0.05) and protein levels of p-NF-*κ*B (*P* < 0.001) and p-I*κ*B*α* (*P* < 0.001) in CUMS rats. Additionally, CUMS rats showed increased protein levels of ASC (*P* < 0.05) and mature Caspase-1 (*P* < 0.05) with slightly upregulated NLRP3 compared with the non-CUMS group (Figures 5(d)–5(f)). Huanglian-Wendan decoction inhibited hippocampal NLRP3 inflammasome activation by markedly reducing protein levels of ASC (11,380 mg/kg: *P* < 0.05) and mature Caspase-1 (5690 mg/kg: *P* < 0.01; 11,380 mg/kg: *P* < 0.001) in CUMS rats. Fluoxetine also inhibited hippocampal NLRP3 inflammasome activation by reducing protein levels of NLRP3 (*P* < 0.05), ASC (*P* < 0.01), and mature Caspase-1 (*P* < 0.01). Consistently, CUMS rats showed an increase in IL-1*β* protein levels (*P* < 0.01), which were ameliorated by Huanglian-Wendan decoction (5690 mg/kg: *P* < 0.05; 11,380 mg/kg: *P* < 0.01) as well as fluoxetine (*P* < 0.05) (Figure 5(g)).

In the hypothalamus, the N/C ratio of NF-*κ*B as well as protein levels of p-NF-*κ*B and p-I*κ*B*α* were observed with a slight increase in CUMS rats. Huanglian-Wendan decoction significantly decreased the N/C ratio of NF-*κ*B (11,380 mg/kg: *P* < 0.05) and protein levels of p-NF-*κ*B (5690 mg/kg: *P* < 0.01; 11,380 mg/kg: *P* < 0.05), but slightly reduced p-I*κ*B*α* protein levels in CUMS rats (Figures 6(a)–6(c)). Fluoxetine showed a nonsignificant effect on the N/C ratio of NF-*κ*B or protein levels of p-NF-*κ*B and p-I*κ*B*α* in the hypothalamus of CUMS rats. NLRP3 inflammasome was found to be activated in CUMS rats as its obviously upregulated component mature Caspase-1 (*P* < 0.05), along with slightly increased protein levels of NLRP3 and ASC. Huanglian-Wendan decoction significantly reduced the protein levels of ASC (11,380 mg/kg: *P* < 0.05) and mature Caspase-1 (5690 mg/kg: *P* < 0.01; 11,380 mg/kg: *P* < 0.001) in CUMS rats (Figures 6(d)–6(f)). Fluoxetine also inhibited NLRP3 inflammasome activation by reducing the protein levels of NLRP3 (*P* < 0.01) and mature Caspase-1 (*P* < 0.05). Consistently, the CUMS procedure induced a significant increase in IL-1*β* protein levels in rats (*P* < 0.01), which were alleviated by Huanglian-Wendan decoction (5690 mg/kg: *P* < 0.05; 11,380 mg/kg: *P* < 0.001) and fluoxetine (*P* < 0.05) (Figure 6(g)).

## 4. Discussion

The present study demonstrated the antidepressant effect of Huanglian-Wendan decoction. Furthermore, Huanglian-Wendan decoction markedly inhibited an inflammatory response in both brain regions and liver of CUMS rats by suppressing NF-*κ*B and NLRP3 inflammasome activation. Of note, the CUMS procedure induced a nonsignificant change in serum IL-1*β* levels, which was indicative of regional inflammation rather than systemic inflammation in depression. Indeed, region-specific alteration of molecules controlling IL-1*β*-related inflammation was also observed within brain regions of CUMS rats, being consistent with our previous report [7]. Although increase in mature IL-1*β* protein levels and the NF-*κ*B pathway (except hypothalamus) and NLRP3 inflammasome activation were observed in all brain regions of the prefrontal cortex, hippocampus, and hypothalamus, molecular changes like p-I*κ*B*α*, ASC, and especially NLRP3 presented variances among different regions. For example, NLRP3 protein levels were significantly increased in the prefrontal cortex, while they were nonsignificantly changed in the hippocampus and hypothalamus of CUMS rats. The prefrontal cortex is mostly reported with NLRP3 inflammasome activation by us [7, 12] and others [10, 11] in CUMS rodents, thus becoming a relatively susceptible brain region in depression. In the present study, nonsignificantly increased NLRP3 protein but significantly increased ASC and downstream activated Caspase-1 and IL-1*β* were detected in the hippocampus of CUMS rats. The NLRP3 protein levels are reported to be increased in the hippocampus of male C57BL/6 mice subjected to 5-week chronic mild stressors [13] and in male 8-week-old BALB/c mice experienced with 4-week chronic mild stressors [14]. These discrepancies might be due to different animal strains and depressed stressor types and durations.

It is known that NLRP3 inflammasome assembly activation requires two steps. First is the priming signals at transcriptional and nontranscriptional levels [32, 33], followed by second signals derived from extracellular ATP, pore-forming toxins, or crystalline materials. Nontranscriptional priming includes posttranslational modifications like the phosphorylation and deubiquitination signals, and even more some metabolites like reactive oxygen species with unknown mechanism [34]. It is possible that some posttranslational priming modifications do not require transcriptional upregulation of NLRP3. Besides, CUMS-induced inflammation, especially neuroinflammation, in the present study should be sterile inflammation, which might show a weaker NLRP3 inflammasome response relative to microbial signals [35]. This weak signal and regional specificity require assay methods with the improved accuracy to verify existing discrepancies of brain NLRP3 protein changes.

The region-specific activation of the brain NF-*κ*B pathway was also observed in CUMS rats in this study. The CUMS procedure caused a slight but significant activation of the NF-*κ*B inflammatory pathway in the prefrontal cortex and hippocampus, but a nonsignificant change in the hypothalamus of rats. Huanglian-Wendan decoction inhibited NF-*κ*B pathway activation in all three brain regions, while fluoxetine failed to significantly alter this pathway in the hypothalamus of CUMS rats. The two drugs showed inhibitory effects on NLRP3 inflammasome activation in the prefrontal cortex, hippocampus, and hypothalamus in CUMS rats, but exhibited a different regulatory feature. Unlike the inhibition of NLRP3 protein in all three brain regions by fluoxetine, Huanglian-Wendan decoction showed nonsignificant inhibition of NLRP3 protein but significant inhibitory potential of ASC and Caspase-1 protein in brain regions, resulting in a similar degree of IL-1*β* reduction as fluoxetine did. Of note, two doses of Huanglian-Wendan decoction exerted a similar antidepressant activity in CUMS rats coinciding with the obvious anti-inflammatory activity as the reduction of IL-1*β* levels in serum, liver, and all brain regions. However, the high dose (11,380 mg/kg) may show better ability to modulate some inflammatory molecules especially in brains than low dose (5690 mg/kg). Unlike the similar inhibition of p-I*κ*B*α* and ACS proteins in the prefrontal cortex by both doses of Huanglian-Wendan decoction in CUMS rats, the low dose failed to affect these two molecules in the hippocampus and hypothalamus, while the high dose showed significant inhibition of p-I*κ*B*α* in the hippocampus and ASC in the hippocampus and hypothalamus. Furthermore, in the liver of CUMS rats, the low dose of Huanglian-Wendan decoction showed a nonsignificant effect on NLRP3 expression while the high dose significantly reduced it. A similar significant inhibition by both two doses of Huanglian-Wendan decoction on hepatic IL-1*β* might result from the significant inhibition on hepatic NF-*κ*B activation and Caspase-1 expression in CUMS rats. These observations indicated that the same final anti-inflammatory performance might result from variant molecular regulations in different tissues, regions, or cells in depression. Besides, the dose-dependent effect of Huanglian-Wendan decoction on modulation of the NF-*κ*B pathway and NLRP3 inflammasome in brain regions might provide mechanical cues of this TCM formula. However, further investigations are needed to elucidate the exact underlying molecular mechanism of antidepression by Huanglian-Wendan decoction.

In TCM theory, *Liver-Qi* stagnation is a possible cause of depression; thus, soothing the *Liver* is a reasonable antidepressant strategy. The liver-brain inflammation axis has been implicated to demonstrate liver inflammation-associated sickness behavior and mood disorders [36, 37], which is supported by our previous study in CUMS rats [12] and others in a quantitative proteomics study of CUMS mice [18]. Huanglian-Wendan decoction is reported to reduce serum IL-6 levels in metabolic syndrome of rats [25]. In this study, although the CUMS procedure induced no obvious change in serum IL-1*β* or IL-6 levels, Huanglian-Wendan decoction significantly reduced both IL-1*β* and IL-6 levels in serum, possibly contributing to its potent hepatoprotection in CUMS rats. These results suggest that Huanglian-Wendan decoction has potential peripheral anti-inflammatory activity, being associated with its antidepression. Thus, blockade of the liver-brain inflammation axis by Huanglian-Wendan decoction could be able to treat depression.

Consistent with its anti-inflammation by Huanglian-Wendan decoction, some of its active compounds have been reported to have anti-inflammatory effects. Berberine, a plant alkaloid in Huanglian-Wendan decoction, is considered the main active compound with several therapeutic bioactivities. For example, it exerts antidepressant-like effects by significantly reducing the immobility time in the forced swim test and tail suspension test in depressed mice [38]. Moreover, berberine suppresses NLRP3 inflammasome activation in monosodium urate crystal-stimulated RAW 264.7 macrophages and rats [39] and in lipopolysaccharide-stimulated RAW 264.7 macrophages and experimental liver injury of mice [40]. Palmatine, another alkaloid component similar to berberine, shows antidepressant-like effects in CUMS mice by reducing brain monoamine oxidase-A activity and serum corticosterone levels [41]. Some flavonoid compounds, such as naringin, hesperidin, and neohesperidin, also exert antidepressant like and/or anti-inflammatory effects. Naringin restores the unpredictable chronic stress-induced depressive behavior and decreases serum TNF-*α* and IL-1*β* levels in mice [42]. Hesperidin shows antidepressant-like effects and reduces prefrontal cortical IL-1*β*, IL-6, and TNF-*α* levels in lipopolysaccharide-treated ICR mice [43]. Neohesperidin decreases hepatic cyclooxygenase-2 and NF-*κ*B expression in paraquat-induced acute liver injury of mice [44]. Therefore, these alkaloid and flavonoid compounds in Huanglian-Wendan decoction may take responsibility for the antidepression mediated by anti-inflammation, which needs further validation.

## 5. Conclusion

In the present study, we demonstrated the antidepressant ability with hepatoprotection of Huanglian-Wendan decoction in CUMS rats coinciding with its obvious anti-inflammatory activity. More importantly, Huanglian-Wendan decoction inhibited a peripheral and central inflammatory response in CUMS rats by suppressing NF-*κ*B and NLRP3 inflammasome activation. These results may provide novel cues of mechanisms and molecular targets mediating the antidepression of Huanglian-Wendan decoction.

## Figures and Tables

**Table 1:** Detailed information of herbs in Huanglian-Wendan decoction.

Chinese name	Botanical name	English name	Part used	Origin (P. R. China)/batch number	Amount (g)
Huang Lian	*Coptis chinensis Franch*	Rhizoma Coptidis	Rhizome	Si Chuan/131219	10
Ban Xia	*Pinellia ternata (Thunb.) Breit*	Rhizoma Pinelliae	Rhizome	Gui Zhou/130610	10
Chen Pi	*Citrus reticulata Blanco*	Pericarpium Citri Reticulatae	Pericarpium	Zhe Jiang/140118	15
Zhi Shi	*Citrus aurantium L*	Fructus Aurantii Immaturus	Fructus	Zhe Jiang/130812	10
Fu Ling	*Poria Cocos (Schw) Wolf*	Poria	Sclerotium	An Hui/131110	20
Zhu Ru	*Bambusa tuldoides Munro*	Caulis Bambusae In Taenias	Cortex	An Hui/131122	15
Gan Cao	*Glycyrrhiza uralensis Fisch*	Radix Glycyrrhizae	Root and rhizome	Nei Meng/131115	3
Sheng Jiang	*Zingiber officinale Roscoe*	Rhizoma Zingiberis Recens	Rhizome	Jiang Su/140805	3

**Table 2:** Identification of the compounds in water extract of Huanglian-Wendan decoction by LC–MS/MS analysis in positive ion mode.

Number	Retention time (min)	Adduct	Extraction mass (Da)	Found mass (Da)	Error (ppm)	Formula	Identification	Reference
1	4.49	+H	265.15467	265.15442	−0.9	C_14_H_20_N_2_O_3_	Subaphyllin	MassBank ID MSJ00004
2	6.58	+	342.16998	342.1702	0.6	C_20_H_24_NO_4_	Magnoflorine	[27]
3	8.69	+H	195.06519	195.06506	−0.7	C_10_H_10_O_4_	Ferulic acid	METLIN ID 4156
4	9.04	+	322.10738	322.10716	−0.7	C_19_H_16_NO_4_	Berberrubine	METLIN ID 86846
5	9.05	+H	597.1814	597.18134	−0.1	C_27_H_32_O_15_	Eriocitrin	METLIN ID 52850
6	9.12	+H	257.08084	257.08056	−1.1	C_15_H_12_O_4_	Liquiritigenin	[28]
7	9.42	+H	289.07066	289.07073	0.2	C_15_H_12_O_6_	Eriodictyol	METLIN ID 3415
8	9.42	+H	597.1814	597.18134	−0.1	C_27_H_32_O_15_	Neoeriocitrin	METLIN ID 52843
9	10.08	+	320.09173	320.09193	0.6	C_19_H_14_NO_4_	Coptisine	[27]
10	10.11	+H	581.18648	581.18619	−0.5	C_27_H_32_O_14_	Narirutin	METLIN ID 52724
11	10.16	+	336.12303	336.12287	−0.5	C_20_H_18_NO_4_	Epiberberine	[27]
12	10.28	+	338.13868	338.13842	−0.8	C_20_H_20_NO_4_	Jatrorrhizine	[27]
13	10.47	+H	273.07575	273.07598	0.8	C_15_H_12_O_5_	Naringenin	METLIN ID 3401
14	10.48	+H	581.18648	581.18619	−0.5	C_27_H_32_O_14_	Naringin	MassBank ID PB005724
15	10.59	+H	579.17083	579.17039	−0.8	C_27_H_30_O_14_	Rhoifolin	METLIN ID 44401
16	10.81	+H	611.19705	611.19675	−0.5	C_28_H_34_O_15_	Hesperidin	METLIN ID 3678
17	11.09	+H	609.1814	609.18146	0.1	C_28_H_32_O_15_	Diosmetin 7-neohesperidoside	METLINID 49249
18	11.17	+H	303.08631	303.08609	−0.8	C_16_H_14_O_6_	Hesperetin	METLIN ID 44508
19	11.17	+H	611.19705	611.19675	−0.5	C_28_H_34_O_15_	Neohesperidin	METLIN ID 3679
20	11.29	+	336.12303	336.12287	−0.5	C_20_H_18_NO_4_	Berberine	Mass Bank ID KO002476
21	11.44	+	352.15433	352.15407	−0.8	C_21_H_22_NO_4_	Palmatine	METLIN ID 6992
22	11.85	+H	419.13366	419.13363	−0.1	C_21_H_22_O_9_	Liquiritin	MassBank ID BML01654
23	12.27	+H	257.08084	257.08056	−1.1	C_15_H_12_O_4_	Isoliquiritigenin	[28]
24	13.21	+H	595.20213	595.20216	0	C_28_H_34_O_14_	Poncirin	MassBank ID PR100363
25	15.04	+H	331.08123	331.08133	0.3	C_17_H_14_O_7_	Tricin	MassBank ID FIO00740
26	16.48	+H	269.08084	269.08099	0.6	C_16_H_12_O_4_	Formononetin	METLIN ID 43917
27	17.60	+H	471.20134	471.20149	0.3	C_26_H_30_O_8_	Limonin	METLIN ID 44458
28	18.19	+H	471.34689	471.34677	−0.2	C_30_H_46_O_4_	Glycyrrhetinic acid	MassBank ID KO003037
29	18.19	+H	823.41106	823.41217	1.3	C_42_H_62_O_16_	Glycyrrhizic acid	METLIN ID 44285
30	18.79	+H	403.13874	403.13865	−0.2	C_21_H_22_O_8_	Nobiletin	METLIN ID 44436
31	20.28	+H	373.12818	373.12836	0.5	C_20_H_20_O_7_	Sinensetin	METLIN ID 49674

**Table 3:** Identification of the compounds in water extract of Huanglian-Wendan Decoction by LC-MS/MS analysis in negative ion mode.

Number	Retention time (min)	Adduct	Extraction mass (Da)	Found mass (Da)	Error (ppm)	Formula	Identification	Reference
1	1.10	−H	191.01973	191.01964	−0.5	C_6_H_8_O_7_	Citric acid	METLIN ID 124
2	1.23	−H	117.01933	117.01934	0.1	C_4_H_6_O_4_	Succinic acid	METLIN ID 114
3	2.95	−H	153.01933	153.01944	0.7	C_7_H_6_O_4_	3,4-Dihydroxybenzoic acid	METLIN ID 3277
4	6.34	−H	353.08781	353.08812	0.9	C_16_H_18_O_9_	Chlorogenic acid	METLIN ID 3498
5	9.46	−H	193.05063	193.05063	0.0	C_10_H_10_O_4_	Ferulic acid	METLIN ID 4156
6	9.86	−H	595.16684	595.16911	3.8	C_27_H_32_O_15_	Eriocitrin	[28]
7	9.92	−H	417.11911	417.11951	1.0	C_21_H_22_O_9_	Liquiritin	[29]
8	10.22	−H	595.16684	595.16911	3.8	C_27_H_32_O_15_	Neoeriocitrin	[28]
9	10.89	−H	579.17193	579.1736	2.9	C_27_H_32_O_14_	Narirutin	[28]
10	11.25	−H	579.17193	579.1736	2.9	C_27_H_32_O_14_	Naringin	[28]
11	11.57	−H	609.18249	609.18517	4.4	C_28_H_34_O_15_	Hesperidin	METLIN ID 3678
12	11.94	−H	609.18249	609.18517	4.4	C_28_H_34_O_15_	Neohesperidin	METLIN ID 3679
13	12.6	−H	417.11911	417.11951	1.0	C_21_H_22_O_9_	Isoliquiritin	[29]
14	13.01	−H	255.06628	255.0665	0.8	C_15_H_12_O_4_	Liquiritigenin	[29]
15	13.92	−H	593.18758	593.18923	2.8	C_28_H_34_O_14_	Poncirin	[28]
16	14.96	−H	271.0612	271.06143	0.9	C_15_H_12_O_5_	Naringenin	METLIN ID 3401
17	15.7	−H	301.07176	301.07191	0.5	C_16_H_14_O_6_	Hesperetin	METLIN ID 44508
18	16.79	−H	255.06628	255.0665	0.8	C_15_H_12_O_4_	Isoliquiritigenin	METLIN ID 44115
19	18.75	−H	821.39651	821.3992	3.3	C_42_H_62_O_16_	Glycyrrhizic acid	METLIN ID 44285
20	28.01	−H	469.33233	469.33327	2.0	C_30_H_46_O_4_	Glycyrrhetinic acid	[29]

**Table 4:** Quantitative parameters of peak area for analysis of components in water extract of Huanglian-Wendan decoction by HPLC.

Name	Peak area of standards (mAU × s)	Peak area of samples (mAU × s)
Ferulic acid	1444.24646	4190.95459
Naringin	1447.29041	9369.6669
Hesperidin	391.07275	5701.23389
Neohesperidin	1659.63416	19228.3
Berberine	3034.30957	2819.72095
Palmatine	2851.47119	8844.96777
Limonin	372.20688	56.03543
Glycyrrhizic acid	17.315	23.85242

**Table 5:** Effects of Huanglian-Wendan decoction on sucrose solution consumption in CUMS rats.

Groups	Duration (week)						
	0	2	4	6	8	10	12
Non-CUMS + vehicle	19.1 ± 0.9	20.0 ± 0.6	20.7 ± 0.8	21.1 ± 1.1	22.5 ± 0.8	22.5 ± 0.6	22.9 ± 0.5
CUMS + vehicle	19.1 ± 1.2	15.8 ± 0.8^###^	16.0 ± 0.6^###^	15.1 ± 0.6^###^	15.4 ± 0.5^###^	15.0 ± 0.7^###^	15.0 ± 0.7^###^
Huanglian-Wendan decoction (5690 mg/kg)	19.1 ± 0.9	14.3 ± 0.8^###^	14.4 ± 0.6^###^	14.7 ± 0.8^###^	19.4 ± 1.1^∗∗^	20.2 ± 1.4^∗∗∗^	21.0 ± 1.3^∗∗∗^
Huanglian-Wendan decoction (11,380 mg/kg)	18.6 ± 0.8	15.4 ± 0.8^###^	14.6 ± 0.6^###^	14.5 ± 0.5^###^	17.8 ± 1.2	18.6 ± 1.2^∗∗^	20.1 ± 1.0^∗∗∗^
Fluoxetine (10 mg/kg)	19.2 ± 0.8	14.5 ± 0.5^###^	15.0 ± 0.4^###^	14.7 ± 0.5^###^	20.2 ± 0.3^∗∗∗^	21.6 ± 0.5^∗∗∗^	21.8 ± 0.7^∗∗∗^

All data were expressed as mean ± S.E.M. (*n* = 12). ^###^*P* < 0.001 compared with the non-CUMS group; ^∗∗^*P* < 0.01 and ^∗∗∗^*P* < 0.001 compared with the CUMS group.
